# Basic and advanced spectrometric methods for complete nanoparticles characterization in bio/eco systems: current status and future prospects

**DOI:** 10.1007/s00216-023-04641-7

**Published:** 2023-03-23

**Authors:** Magdalena Borowska, Krzysztof Jankowski

**Affiliations:** Chair of Analytical Chemistry, Faculty of Chemistry, Warsaw University of Technology, Noakowskiego 3, Warsaw, 00-664 Poland

**Keywords:** Nanoparticle characterization, Spectrometric techniques, Single-particle analysis, Chemical composition, Surface analysis, Synthesis yield

## Abstract

The use of engineered nanoparticles in the environment and human life has increased in the last 20 years. The risk assessment concerning application of nanomaterials in biological systems requires their thorough characterization. Understanding the correlations between physicochemical properties of nanoparticles concerning not only the size, particle size distribution, number concentration, degree of aggregation, or agglomeration but also solubility, stability, binding affinity, surface activity, chemical composition, and nanoparticle synthesis yield allows their reliable characterization. Thus, to find the structure-function/property relationship of nanoparticles, multifaceted characterization approach based on more than one analytical technique is required. On the other hand, the increasing demand for identification and characterization of nanomaterials has contributed to the continuous development of spectrometric techniques which enables for their qualitative and quantitative analysis in complex matrices giving reproducible and reliable results. This review is aimed at providing a discussion concerning four main aspects of nanoparticle characterization: nanoparticle synthesis yield, particle size and number concentration, elemental and isotopic composition of nanoparticles, and their surface properties. The conventional and non-conventional spectrometric techniques such as spectrophotometry UV-Vis, mass spectrometric techniques working in conventional and single-particle mode, or those based on optical emission detection systems are described with special emphasis paid on their advantages and drawbacks. The application and recent advances of these methods are also comprehensively reviewed and critically discussed.

**Graphical abstract:**

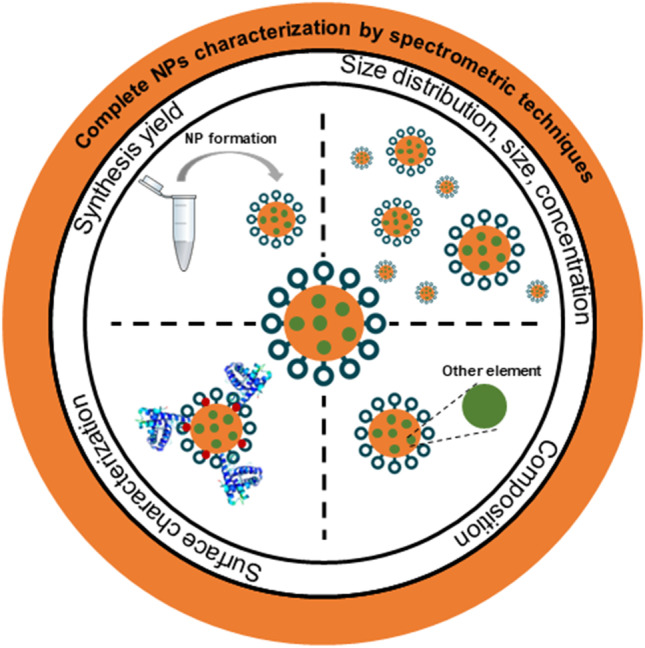

## Introduction

Nanotechnology is one of the most rapidly growing field of science in recent years. Due to the unique physicochemical properties compared with bulk materials, nanomaterials have found applications in various fields including biomedical and environmental applications [1–3]. Materials in nanosized scale can occur in the environment and human life not only as a results of bioformation but also can be produced unintentionally during natural process or intentionally for specific applications [4]. According to the European Commission recommendation, a nanomaterial is a natural, incidental, or manufactured material containing particles in an unbound, aggregate, or agglomerate form, where more than half or more of the particles present in particle population have one or more external dimensions ranging from 1 to 100 nm [5, 6]. Nanoparticles (NPs) pose a potential risk to human health and environment, thus challenging scientists and engineers in multiple ways, from NP characterization and fate in complex matrices to NP effects in natural systems [7–9]. In the natural systems, NPs can undergo a number of physical and chemical transformations such as dissolution and release ionic species to the media, and creation of agglomerates or aggregates [10, 11]. The changes in NP physicochemical properties are thought to have significant influence on their altered behavior. In addition, some differences in physical, chemical, and toxicological properties between NPs and larger-sized particles were observed [12–14]. 

The toxicity effect of NPs and their potential application in biological and environmental systems depend on several factors such as size, chemical composition, structure, and NP synthesis yield. The understanding of NP surface activity enables for recognition of NP functionality as well [15]. All of them are crucial in toxicological and environmental studies, regulatory control, and quality assessment [16]. Thus, the identification and comprehensive physicochemical characterization of NPs in various types of biological and environmental media, including samples with a complex matrix, enable us to better understand NP behavior. In comparison with bulk materials, analysis of NPs is sometimes difficult because of too small size and low quantity [9, 16, 17]. Consequently, a comprehensive approach, by combining reliable methods and techniques in a complementary way, is required.

The NP properties can be characterized by different microscopic and spectroscopic techniques [18, 19]. Although various microscopic techniques are a basic analytical tool for nanomaterial characterization, they exhibit some limitations, including tedious sample preparation and low reliability of the results due to the limited sample amount used and the relatively low statistical significance of data examined. Moreover, a time-consuming sample preparation based on a drying process may lead to aggregation of NPs, especially in environmental samples [7, 9, 19]. Clearly, alternative approaches are needed. Among different analytical tools for the identification and quantification of NPs, spectrometric techniques offer outstanding capabilities. The detailed composition of NPs, their characterization concerning size and surface properties, and the presence of dissolved ions in NP suspension could be investigated by spectrometric techniques such as UV-Vis spectrophotometry [20, 21], atomic absorption spectrometry (AAS) [22, 23], inductively coupled plasma mass spectrometry (ICP-MS) [24, 25], inductively coupled plasma or microwave induced plasma optical emission spectrometry (ICP or MIP-OES) [26, 27], and inductively coupled plasma time-of-flight mass spectrometry (ICP-TOF-MS) [28, 29]. Spectrometric techniques, where nanoparticle is considered as analyte, could provide elemental and molecular information of the NPs examined. The analyzed NP-containing samples can be subjected to both the analysis of the entire NP population and the analysis of individual nanoparticles, particle by particle. Compared with conventional analysis of total analyte content, single-particle (SP) analysis provides many new and detailed information such as particle size and composition of each single NP as well as the NP number concentration [19].

**Fig. 1:**
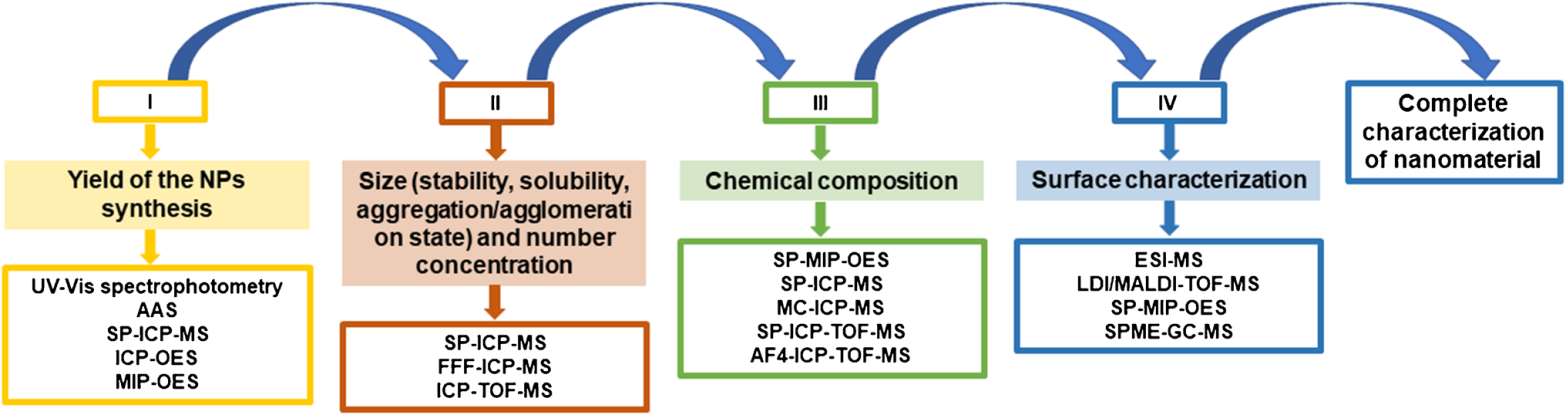
Summary of the general milestones and selected complementary methods applied for the NP complete characterization

In the recent years, several reviews concerning the application of analytical methodologies for nanoobjects have been published. These papers mainly focused on the use of mass spectrometry techniques for detection and quantification of NPs in different types of samples [1, 4, 7, 16, 17, 19, 30, 31]. The main aim of this paper is to critically review the spectrometric approaches for NP characterization studies. The NP characterization using spectrometric methods gives the big picture of nano-size material safe use, where four main targets can be designated as presented in Fig. 1: determination of the yield of NP synthesis (I), size (stability, solubility, aggregation/agglomeration state) and number concentration determination (II), investigation of chemical composition (III), and surface characterization (IV). Each of the mentioned tasks should be carefully designed and performed with the incorporation of proper control samples. In this review, advantages and limitations as well as the influence of the chosen method on the qualitative results will be provided. Moreover, the principles of reviewed techniques and the current problems with sample preparation before analysis will be briefly discussed.

## Determination of the yield of NP synthesis

A properly designed synthesis of NPs, using non-toxic reagents and characterized by high synthesis yield without compromising the functionality of final product, increases the chance of application of synthesis product in biological and environmental systems [32–34]. Concerning these applications, NPs were usually produced in aqueous media, where metal ions are reduced to form NPs with subsequent charge stabilization by adsorption of different species to suppress aggregation, thus resulting in a stable colloid [35]. Since nanoscale metal particles and metal ions may induce independent or combined toxic effects, it is important to know whether the natural system is exposed to NPs, ions, or both [36, 37]. It should be mentioned that in the natural environment, the organisms are always exposed to the composition of all forms of the analyte. The addition of contaminated NPs which are used for a plethora biomedical applications, such as cellular therapy, tissue repair, drug delivery, or bioseparation processes may enhance the toxic effect. Actually, the determination of the yield of synthesis considered as the conversion rate of reagents into NPs [34] requires analytical tools to achieve complete characterization of synthesis process and determination of free form (dissolved) of analyte. Thus, the determination of the total concentration of ionic precursor in the post-reaction NP suspension is important for the assessment of both the yield of synthesis and the toxicity level of the end product. Alternatively, synthesis yield can be considered as an amount of final product obtained after its isolation and purification [16, 34]. However, most purification methods are batchwise and time-consuming. The purification process could fail to remove unwanted molecules or may result in NP material loss within, for example, chromatography separation or centrifugation. According to that, the highest possible synthesis yield is desired. A summary of the advantages and disadvantages of the discussed analytical methods dedicated to determination of the synthesis yield is presented in Table 1.

**Table 1:** Summary of the different analytical methods for NP synthesis yield determination discussed in the text

Analytical technique	Type of analyte	Advantages	Disadvantages
UV-Vis spectrophotometry	NPs	Monitoring of NP formation	Limited to the comparative analysis
AAS	Dissolved ions or ions from NP fraction	Simple total element concentration determination with good sensitivity	Sample preparation may lead to loss information regarding the NP size
ICP-OES			
ICP-MS			
PCVG-MIP-OES	Dissolved ions	Determination of unreacted ions directly in the reaction mixture	Limited to the analysis at ppm levels
SP-ICP-MS	NPs, dissolved ions	Simultaneous determination of NP size/number concentration and concentration of dissolved ions; direct determination of the yield of synthesis	Difficulties in discrimination between the NP and dissolved metal form

The yield of NP synthesis could be monitored using UV-Vis spectrophotometry and estimated as the area under the curve of the UV-Vis absorbance spectrum recorded for the evaluated nanoparticles [21]. The formation of NPs is often confirmed through UV-Vis absorption spectrum at their characteristic wavelengths. Also, UV-Vis spectrophotometry was combined with AAS for the elemental characterization of colloidal dispersion of silver NPs (AgNPs) and to confirm the synthesis yield [21, 22, 38]. Using AAS, Quintereo-Quiroz et al. determined total silver concentration in the AgNP suspension after synthesis in order to determine the yield of synthesis and to optimize the synthesis conditions [21]. In turn, hydride generation AAS was applied to monitor changes in the concentration of toxic selenium oxyanions added to the culture medium during biosynthesis of sulfur-selenium (Se^0^S^0^) NPs by bacterial *Azospirillum brasilense* strain, thus evaluating detoxification efficiency [39]. Concerning the synthesis yield, the monitoring of metal concentration is especially interesting for multi-elemental nanostructures, thus enabling the evaluation of the relative concentration of co-existed metals in the nanoparticles and the investigation of their structural evolution during the synthesis. For the purpose of characterizing gold nanorods synthesized by using the surfactant cetyltrimethylammonium bromide as a stabilizer and shape-directing agent, and gold-silver core–shell NPs which present a high content of polyvinylpyrrolidone acting as a stabilizer, Godoy et al. determined the gold content in NPs and other gold species (derived from unreacted precursor) in colloid [40]. After the fractionation and purification of NP colloid by centrifugation, a microwave-assisted digestion with a mixture of hydrochloric, nitric, and sulfuric acids was applied prior to the gold quantification by ICP-MS and ICP-OES. The authors found out a high synthesis yield of almost 100%, indicating that no gold remains as free ions in the post-reaction colloid, in colloid after washing, and in colloid before the growth of the silver shell.

The synthesis yields conducted by conventional spectrometric techniques mentioned above are calculated from the results of the determination of dissolved ion concentration and element concentration in the NPs and by using the known initial concentration of element ions at the start of the synthesis [22]. However, the determination of NP synthesis yield required isolation of NP particles from the background of dissolved ions [41]. In order to distinguish between NPs and dissolved forms of element, these fractions could be separated by offline physical methods such as ultracentrifugation, ultrafiltration, and dialysis, but problems arise from sorption of different elements on membranes and long running times are required for separation and determination of different element-species [32]. Luo et al. proposed a co-precipitation method to preferentially adsorb silver anions in mixtures containing AgNPs by magnetic graphene oxide [42]. Although the selectivity of the adsorbent required further improvement, this method showed great potential both to determine synthesis yield in the NP suspension and to improve particle sizing by SP-ICP-MS. Apart from conventional physical methods for quantification of the ions in NP fraction, extraction could be performed offline or online [24, 36, 43, 44]. Huang et al. [45] proposed the fractionation of ionic and nanoparticulate species by using ultra-centrifugal units. However, the method was used for preconcentration and speciation, namely distinguishing dissolved ions and NPs in biological and environmental samples, not for the determination of the yield of synthesis.

**Fig. 2:**
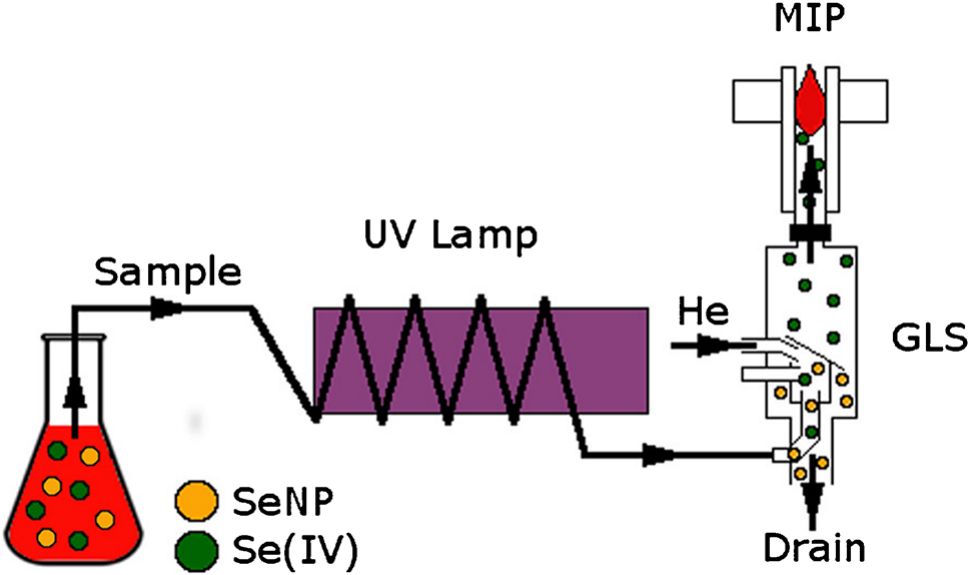
Schematic representation of the PCVG-MIP-OES system. Reproduced from Bartosiak et al. [27] with permission from Elsevier (Copyright Clearance Center’s RightsLink service®)

The analysis of samples containing NPs to determine both converted and unreacted ions usually requires sample preparation step. However, little has been published about the analytical monitoring of NP synthesis by direct analysis of the reaction mixture. Green analytical procedure suitable for direct monitoring of selenium nanoparticle (SeNP) synthesis characterizes the SeNPs produced by non-toxic reagents and determines the yield of the reaction which was proposed by Bartosiak et al. [27]. They applied photochemical vapor generation (PCVG) technique coupled with MIP-OES for selective determination of unreacted selenium(IV) oxyanion in the presence of SeNPs and matrix components, without the need of NP separation. The proposed method, based on the conversion of unreacted ions into volatile species under UV irradiation in the presence of 15% (v/v) acetic acid, could be likely applied for determination of SeNP synthesis yield in order to assess the NPs’ overall safety and toxicity. Figure 2 presents a schematic diagram of PCVG-MIP-OES system for determination of SeNP synthesis yield.

Usually, either NPs or dissolved ion concentration was determined after digestion procedure. During digestion, NPs were converted into their ionic form [41]. Thus, the use of concentrated acids, high temperature, and pressure in the digestion procedure caused loss of the information regarding the size of the NPs. As an alternative method to achieve complete information about synthesis yield and characterize the size distribution of nanoparticles, single-particle inductively coupled plasma mass spectrometry (SP-ICP-MS) could be applied [46]. The presence of dissolved species in sample leads to production of the constant baseline, whereas the NPs give rise to individual signal events [30, 47]. SP-ICP-MS is an analytical method with the potential to provide also the quantitative information about the number and mass concentrations, and about the mass of element/s per NP or NP size [30]. However, the threshold of this method involves discrimination between the NP and dissolved metal forms. In this case, the dissolved ions produce a constant continuous signal that overlaps the NP signal, which can prevent its identification, especially for smaller NP sizes [48]. Thus, SP-ICP-MS could be applied to determine the synthesis yield in a situation where there is a clear distinction between the full particle size distribution and the baseline signal, as is in the case with 60-nm gold NPs (AuNPs) suspended in ultrapure water. The cut of baseline signal corresponds to dissolved ionic fraction as well as it defined the NP size limit of detection (LOD). Thus, even if mathematical approach to calculate threshold value is applied [17], the NP fraction with diameter smaller than size LOD is included in dissolved ions.

The optimization of synthesis conditions for maximum amount of produced NPs is often included in the synthesis method development. However, it is not a standard practice to publish synthesis yield with each new synthesis protocol, even if the end product is applied in natural systems. As an example, Uson et al. could not compare the productivity of their microreactor, used for synthesis of ultrasmall superparamagnetic iron oxide nanoparticles (SPIONs), against publications from other laboratories because the synthesis yield was not reported in the literature [49]. It makes a significant problem for evaluating NP syntheses.

## Determination of the particle size (stability, solubility, aggregation/agglomeration state) and particle number concentration

The most common characteristics examined by SP-ICP-MS and reported in the recent papers concerning NP analysis in biological and environmental samples, such as tissues and plants, were particle size distribution, mean/median particle size, and particle number concentration [7, 50]. Importantly, the core size of NPs determined by SP-ICP-MS is calculated from the element masses assuming the ideal particle geometry and a known density, and sometimes, it is far from the real particle size. The other aspects affecting the trueness of the results are, for example, matrix/plasma polyatomic interferences, physicochemical form of analyte, or the size of NP, which may result in less efficient atomization or ionization of NPs in plasma [30, 51]. Moreover, there is still a lack of multiple matrix standard reference materials to perform accurate particle size and particle number concentration calibrations [50]. Thus, confirmation of the obtained results by independent method (for example, microscopic techniques) is recommended. It should be mentioned that discrepancies in sizing of organic coated nanoparticles by transmission electron microscopy (TEM) and SP-ICP-MS were observed. For example, Barber et al. reported diameter values obtained by TEM for polystyrene brush-coated AuNPs higher than those obtained by SP-ICP-MS [52]. The most studies of NP size and size distribution rely on the measurement of a single isotope of analyzed element characterized by the relatively low impact of spectral interferences and the highest signal to noise ratio. Thus, when polydisperse nanomaterial is studied, the larger particles can produce signals outside the detector linear range. Bucher et al. proposed the use of combining data from two Ti isotopes, to fully characterize size distribution of polydisperse food-grade titanium dioxide NPs (TiO_2_ NPs) [53]. Interestingly, the analytical platform consisting of flow field-flow fractionation inductively coupled plasma mass spectrometry (FFF-ICP-MS) and SP-ICP-MS proved useful for the study of shape transformation between silver nanospheres and silver nanoplate based on their sizes [54]. Phanwichean et al. [54] evaluated the changes in size distribution of NPs after inducing shape transformation from nanosphere to nanoplate by redox reaction using hydrogen peroxide. They observed the increase of particle diameter and broader size distributions with the bimodal characteristics when nanoplates were formed. The opposite conclusions were reported for nanoplates transformation to nanospheres induced by bromide potassium addition. Interestingly, the authors proposed the combined use of FFF-ICP-MS, SP-ICP-MS, and TEM to estimate the layer thickness of NP stabilizing agent, edge length, and plate thickness of silver nanoplates.

To study complex samples of inorganic particles modified with organic compounds, FFF technique can be used to separate particles according to their hydrodynamic size. In the work of [55], this technique was coupled with ICP-TOF-MS working in standard and SP mode to determine the hydrodynamic and core sizes, size distribution, elemental composition, and number concentration of micro-alloyed steel particle determination. Compared to single-particle inductively coupled plasma quadrupole mass spectrometry (SP-ICP-QMS), it does not use a magnetic, electrostatic, or radio frequency (RF) field to disperse or filter ions for individual *m/z* ion detection. In this state-of-the-art technique, the generated ions pass through a transfer and focusing region and enter to mass separate system, the TOF mass analyzer, where the packet of ions generated from individual NP are extracted simultaneously, accelerated, and reach the detector depending on the type of ions. The flight time of ions is a function of the mass at the same flight distance [4, 56]. Von der Au et al. proposed the combination of a microdroplet generator and an ICP-TOF-MS for the fast and reliable size determination of platinum NPs (PtNPs) in different matrices, i.e., sea water [57].

Colloidal stability, as well as aggregation or agglomeration and dissolution stage of NPs, affects the biological interaction, toxicity, and environmental fate of NPs [58–60]. An ideal method would analyze aggregation and dissolution in situ [61]. These processes can be quantitatively examined in a simultaneous or separate way. Donahue et al. proposed the use of SP-ICP-MS as an unbiased high-throughput analytical technique to quantify NP aggregation with single-aggregate resolution in situ [62]. Generally, the aggregates are clusters of multiple individual NPs and they are detected by the SP-ICP-MS as a single event exhibiting a mass that corresponds to a single-nanoparticle mass multiplied by the number of NPs per aggregate. However, this method requires NPs with narrow mass distribution, hence narrow size distribution, which typically is not the case for the NPs present in the biological and environmental samples. The analysis of aggregates and colloidal stability of NPs in the biological and environmental matrices requires efficient extraction of NPs from sample material. It should be mentioned that some sample preparation protocols could affect NP behavior and can change the state of NPs due to their aggregation or dissolution that implies the loss of some particle fractions [63]. Huang et al. [45] compared the alkaline and enzymatic sample preparation protocols used to release ionic Ce and ceria NPs (CeO_2_ NPs) from animal tissues. They concluded that after alkaline extraction using tetramethylammonium hydroxide (TMAH), the formation of Ce-containing precipitates was observed, and the calculated CeO_2_ NP mass concentration and particle number concentration values were overestimated due to the increased transport efficiency in SP-ICP-MS by pretreated matrix at low dilution level. They suggested that the presence of TMAH and organic carbon species after sample preparation suppressed the surface tension, resulting in smaller droplets generated in the nebulizer. Interestingly, Xu et al. [64] used the agglomeration process of AuNPs to accomplish the sensitive detection of target hepatitis B virus DNA by SP-ICP-MS. In this research, the AuNPs smaller than the detectable size of ICP-MS were employed as the elemental tags. The presence of NPs smaller than the minimum detectable size in the analyzed sample caused that a low and stable baseline would occur in the recorded time scan. After addition of the target analyte, AuNP probes agglomerated, and the pulse signal of Au was easily distinguished.

It should be mentioned that the SP-ICP-MS analysis requires a multiple dilution of NP suspension to avoid the presence of several particles in the plasma at the same time. However, the dilution of NPs in water leads to destabilization due to aggregation or agglomeration [65]. In the real, biological and environmental, samples, the NPs are expected to be found at low concentration. Moreover, some of NPs are highly soluble under environmental conditions [61, 66]. Thus, they are measured in the presence of high background coming from the dissolved metal. Fréchette-Viens et al. [67] improved the size detection limits for the soluble zinc NPs (ZnO NPs) of ca. 14.3 nm in river water and 17.7 nm in rainwater, thanks to the application of an ion exchange column (IEC) and a sector-field ICP-MS as well as short dwell time (50 µs). The limitations concerning sensitivity of quadrupole analyzer hinder the analysis of particles below 10 nm. Even if short dwell time is applied, the available dynamic range of ICP-MS is limited. Shaw et al. [68] proposed the use of magnetic sector ICP-MS with GHz ppm^−1^ sensitivities, smaller dwell time (down to 10 µs) combined with automatic variable width peak integration for analysis of AuNPs smaller than 10 nm and more concentrated than for traditional SP-ICP-QMS. The described technique improved both the signal to background ratio and dynamic range. However, to distinguish between NPs and dissolved ions, improvements in both instrumentation and data processing are required [17, 30, 69].

Although the recent studies presented the use of other spectrometric techniques, i.e., AAS, to characterize NPs for the quantification of AgNPs [70] and speciation of Zn^2+^ and ZnO nanoparticles [71], the information concerning size distribution or the particle number concentration was lost during the analysis and sample preparation.

Table 2 lists some examples of NP analysis in biological and environmental samples discussed in the the “Determination of the particle size (stability, solubility, aggregation/agglomeration state) and particle number concentration” section.

**Table 2:** The selected examples of NP characterization concerning their size and concentration in biological and environmental samples

Analytical technique	Type of analytes	Matrix	Type of measurand	LOD_size_	Reference
SP-ICP-MS	Ionic Ce CeO_2_ NP_S_	Animal tissue	Particle number concentration and size distribution	15 nm	[45]
SP-ICP-MS	SeNPs	Yeast	Particle number concentration and size distribution	18 nm	[51]
SP-ICP-MS	Polydisperse TiO_2_ NP_S_ (^47^Ti and ^48^Ti)	Food	Particle size distribution	^47^Ti 67–85 nm ^48^Ti 28–36 nm	[53]
SP-ICP-MS FFF-ICP-MS	AgNPs as nanospheres and nanoplates forms	Directly after formation	Particle size distribution to monitor particle transformation	-	[54]
SP-ICP-TOF-MS	Nb and TiNb carbonitride (CN) NPs	Micro-alloyed steel	Particle number concentration, size distribution, presence of agglomerates, multi-elements analysis	NbCNNPs—28 nm TiNbCNNPs—45 nm	[55]
SP-ICP-MS	Hepatitis B virus DNA labeled AuNP	Human serum	Frequency signals appeared after agglomeration of AuNPs	-	[64]
IEC-SP-ICP-MS	ZnO NPs	River water and rainwater	Particle number concentration and size distribution	14.3 nm in river water and 17.7 nm in a rainwater	[67]
Dispersive suspended microextraction followed by oxidative dissolution back-extraction and AAS	Ag in AgNPs	Bottled water, river water, effluent wastewater	Selective extraction of AgNPs and total concentration of AgNPs	-	[70]
Solid sampling high-resolution continuum source electrothermal AAS	ZnO NPs, Zn^2+^, and total Zn	Cosmetics	Speciation analysis	-	[71]

## Determination of the elemental and isotopic composition of NPs

The emerging growth in the development, production, and application of multi-element engineered NPs (ENPs), which are widely incorporated into commercial products, requires advanced analytical methods which allow for size fractionation and chemical quantification of nanomaterials [72, 73]. Some of the published studies regarding determination of the elemental and isotopic composition of NPs are summarized in Table 3.

**Table 3:** The examples of spectrometric techniques for NP elemental/isotopic composition determination

Analytical technique	Purpose of the analysis	Analytes	Reference
SP-MIP-OES	Elemental composition	ZnO, MgO, In_2_O_3_, Fe_3_O_4_, SnO_2_, and SiO_2_ NPs	[74]
		SeNPs and SeNPs-HSA conjugate	[75]
SP-ICP-MS	Dual-mass measurements on individual particles	AgNP and AuNP	[79]
SP-ICP-MS	Estimation the core and shell thickness of bimetallic nanoparticles	Ag-Au NPs	[82]
MC-ICP-MS	Isotopic signatures	Hg in HgSeNPs	[83]
MC-ICP-MS	Isotopic composition	Iridium-osmium NPs	[85]
MC-ICP-MS	Isotopic composition	AgNPs	[86]
SP-ICP-TOF-MS	Classification of engineered, incidental and natural NPs based on elemental composition	Ce-NPs	[89]
SP-ICP-TOF-MS	Classification of engineered, and natural NPs based on elemental composition	Ce-NPs	[90]
SP-ICP-TOF-MS	Composition, size distribution, and concentration	BiVO_4_, (Bi_0.5_Na_0.5_)TiO_3_, and steel (which contains Fe, Cr, Ni, Mo) NPs	[91]

**Fig. 3:**
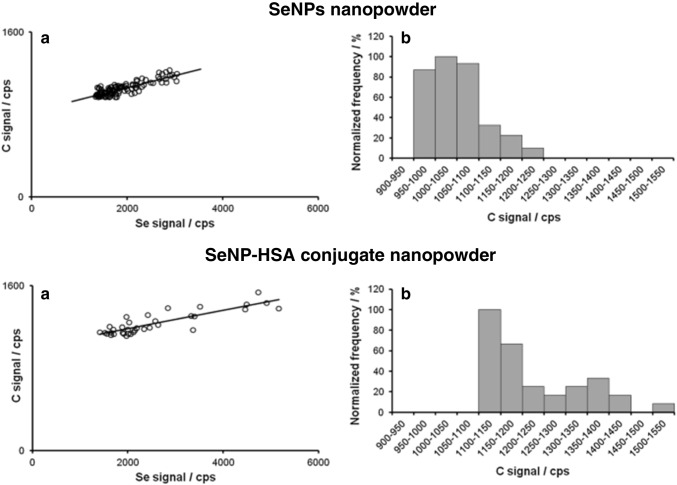
**a** Correlations between C and Se events for selenium nanopowder and SeNP-HSA conjugate nanopowder; **b** carbon signal intensity distribution plot for SeNPs and SeNP-HSA conjugate nanopowder; reproduced from Borowska et al. [75] licensed under a Creative Commons Attribution 4.0 International License (http://creativecommons.org/licenses/by/4.0/)

Borowska et al. proposed new MIP-OEStechnique operating in a single-particle mode for characterizing powder nanomaterials [74]. In this technique, nanopowders were introduced to a helium plasma by pneumatic nebulization based on fluidized bed approach and measured with a time resolution of 4–20 ms. This technique was applicable to the multi-element detection of both metals and non-metals advantageously in simultaneous mode. The nanopowder composition was evaluated by examining the synchronicity of pulses recorded for each element of interest where those being synchronous correspond to one particle only. Based on the plot of the signal intensity correlation recorded for all synchronous pulses, it is possible to confirm the reproducibility of elemental composition or even stoichiometry of the examined compound. It should be noted that the proposed technique cannot be considered as a mature methodology due to the lack of calibration work. Nevertheless, this technique was applied for characterization of biogenic SeNPs synthesized using yeast extract [75]. The yeast extract contained biomolecules, with carbon in their structure, which could functionalize NP surface. The light emission from carbon atoms was used as a marker for identification of functionalizing groups and determination of elemental composition of NPs. Thus, the emission from Se and C was measured with a time-resolved manner, where each pulse corresponds to one particle only. The correlation between Se and C signals recorded for all particles in SeNP samples (a) and SeNPs conjugated with human serum albumin (HSA) samples (b) presented in Fig. 3 showed that the signals were synchronous. However, after interaction of SeNPs with HSA, the correlation between recorded signals worsens from 0.7391 to 0.6449 and the increase of shift factor was observed. The latest occurred due to the higher number of C atoms per SeNP. Thus, obtained results confirmed that each NP was covered by molecules containing carbon atoms and the amount of functionalized groups corresponded with NP size (Fig. 3).

Although OES detection system is a powerful tool for single-particle multi-element analysis, it cannot be used for isotope detection [76–78]. SP-ICP-MS technique enables rapid measurements of size, mass, concentration, and element/isotope concentration. However, the monitoring of multiple elements in an individual NP in a single run is not possible [4, 72, 78]. Although the increase in acquisition time and peak-jumping enables for multi-element analysis of single particle to some extent, it was observed that the multiple detection is still limited to at most two targeted elements in each NP [69]. The recent study performed dual-mass measurement of individual particles using quadrupole-based ICP-MS (ICP-QMS) [79]. This type of detection is usually called as “quasi-simultaneous” analysis of NP composition concerning 2 elements/isotopes due to the high frequency of time-mass scanning. Nevertheless, the lack of acquisition data in long settling time, i.e., time separating the measurements of two consecutive masses which corresponds to time required for stabilizing the quadrupole, leads to loss of signal and measurement of incomplete events [4, 79–81]. Concerning settling time, in order to allow sufficient value for the quadrupole, the analysis should be focused on the detection of two isotopes with close *m*/*z* ratio, and where the difference between two isotopes should be smaller than 20 mass units [79]. Heetpat et al. applied SP-ICP-MS with simultaneous dual-mode detector for bimetallic NP analysis to determine Au core size and Ag shell thickness [82]. It was demonstrated that it is possible to differentiate between Ag-Au bimetallic NPs and core-shell Ag-Au NPs. This study based on observations of average signal profiles and calculation of the molar ratio of both components with the possibility of figuring out which of the two element comprises the core.

The isotopic composition, as well as elemental composition, of NP plays an important role in NP characterization [83]. Isotopic ratio data for individual particles are beneficial for biological and environmental studies dealing with their source identification. The technique which can be useful to determine the isotopic ratio of individual NP is multi-collector inductively coupled plasma mass spectrometry (MC-ICP-MS) with a magnetic analyzer and multiple-collector array using Faraday detectors [4, 84]. Briefly, different *m/z* ions move with different radii in the homogeneous magnetic field under the action of Lorentz force, and then, they reach different collectors [4]. Hirata et al. showed that the MC-ICP-MS system with a high-time resolution data acquisition could be used to Os isotopic ratio measurements from single NP [85]. In this study, the simultaneous data acquisition of four isotopes was acquired with a time resolution of up to 10 µs, which permitted the quantitative analysis of four isotopes to be carried out from transient signals produced by the NPs. However, the proposed system was not user-friendly due to the careful calibration of detector gains required. The other study presented by Yamashita et al. demonstrated the capability of MC-ICP-MS for particle analysis by determining isotope composition for individual AgNP [86]. They reported that the measured isotope ratios were consistent with the criterion value with a relative deviation between 1.76 and 0.33% for 40 and 100 nm, respectively. The recent study showed the benefits in modification of the detection in MC-ICP-MS instrument with timestamp digitization of all single ion detection events to 0.5 ns accuracy and with no predetermined integration window [87]. The ion arrival time, which was much shorter compared with ionic species introduced into the plasma, could be used to discriminate particle signal from background. This approach was applied to characterize, i.e., AuNPs. MC-ICP-MS allows simultaneous detection of multiple isotopes and can become a powerful tool for monitoring elemental and isotope ratios from NPs of multiple components in a particle-by-particle mode. However, MC-ICP-MS is not adapted for multiple element analysis as it can only target a narrow range of *m*/*z* in one data acquisition cycle [85]. Moreover, a low amount of sample and/or a low target isotope concentration can strongly compromise applications focused on high-precision isotopic analysis of nanoparticles by MC-ICP-MS, and the widespread application of MC-ICP-MS is limited due to its high cost and enormous size [4, 69].

To examine elemental composition of single particle by detection of multiple isotopes, ICP-TOF-MS can be used, providing isotopic masses in particles [28, 88]. The elements consisting of single particle can be distinguished based on the differences in the time required to reach the detector [4]. Several studies used single-particle inductively coupled plasma time-of-flight mass spectrometry (SP-ICP-TOF-MS) for the characterization of NPs in environmental and biological matrices. The researchers identified that the large amount of data and information obtained using mentioned technique makes the results’ interpretation very challenging [72]. Thus, in recent years, SP-ICP-TOF-MS emerged as a useful method for classification of NP type based on multi-element fingerprinting using machine learning algorithm such as the hierarchical agglomerative clustering [29, 89]. This technique was applied, for example, to characterize elemental composition of road dust NPs, which were further identified based on TEM coupled with energy-dispersive X-ray spectroscopy and selected area (electron) diffraction techniques [29]. Praetorius et al. [90] demonstrated the applicability of single-particle multi-element fingerprinting method to distinguish between engineered CeO_2_ NPs and natural Ce-containing NPs in soils at environmentally relevant concentrations. On the other hand, the recent study presented the coupling of asymmetrical flow field-flow fractionation (AF4) with ICP-TOF-MS working in standard and SP mode to determine elemental composition. Compared with conventional SP-ICP-TOF-MS technique, the coupling with AF4 provided additional and complementary information including the differential of elements distributions for smaller and larger particles. However, some aspects of the online coupling with AF4 such as an optimal particle number concentration need to be investigated [55]. Moreover, the ICP-TOF-MS technique was used for the multi-element analysis of composite commercial core-shell NPs [91]. The TOF instruments showed ability to quantitatively determine the composition of multi-element NPs. It should be noted that the currently available reference materials of NPs are dominated by engineered mono-elemental NPs [1]; thus, the applicability of SP-ICP-TOF-MS to analysis of NP elemental composition in real samples containing different types of matrix (such as biological fluids) needs to be further investigated.

## Characterization of the NP surface

**Fig. 4:**
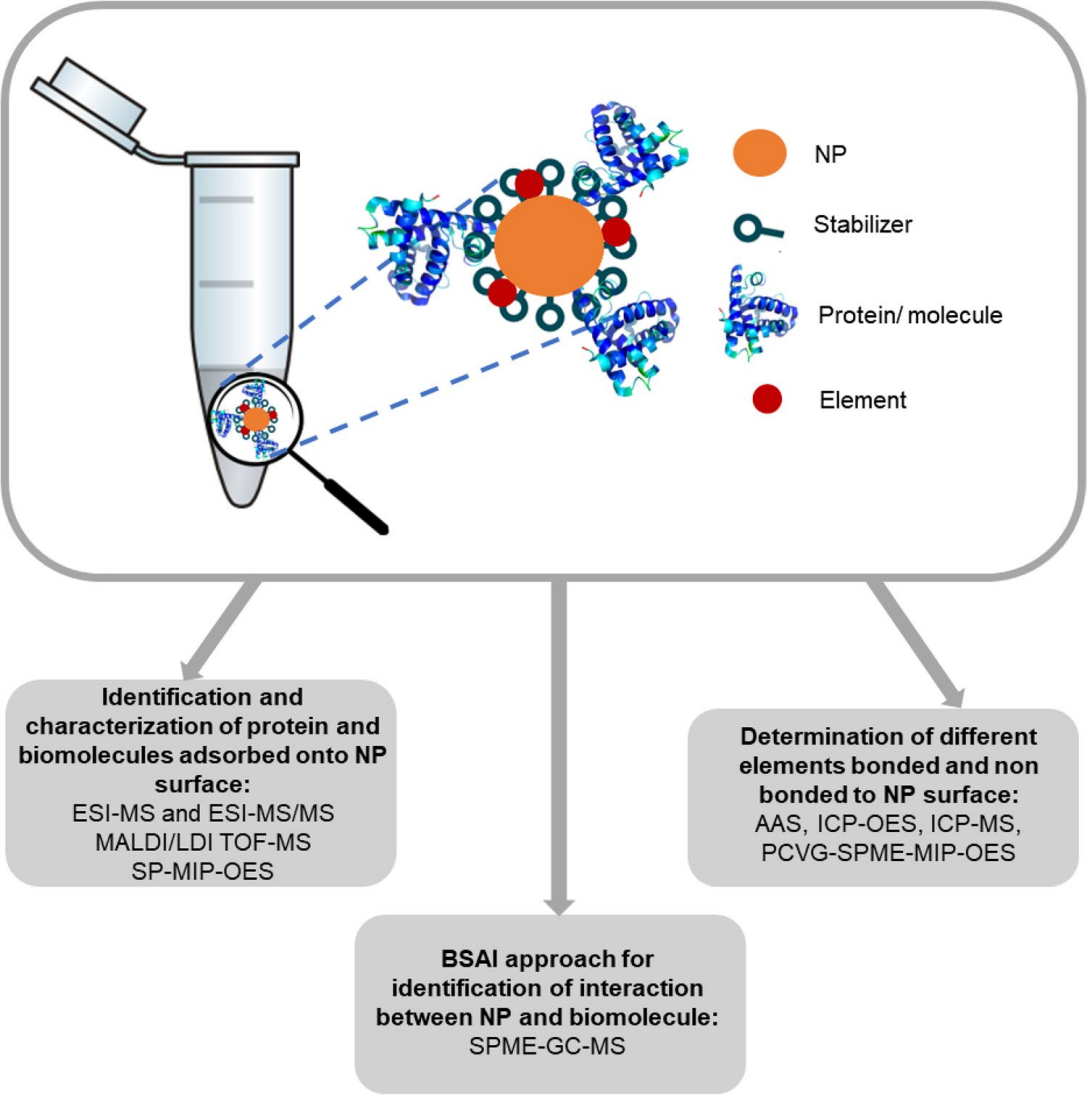
Summary of the methods applied for the NP surface characterization

The reactivity of NPs in biological and environmental systems strongly depends on the properties of their surface [92]. Thus, those are crucial attributes for many of their diverse applications due to correlation between stability, activity, therapeutic efficacy, and toxicity of nanomaterials with surface characteristics [15, 93]. NPs can be functionalized with a variety of molecules and the structure characterization of their surface regarding conjugation chemistry and ligand identity is a crucial point in the development of functionalized NPs with diverse applications [94]. As a result, in order to understand and control particle behavior and properties, the analysis of NP surface is often essential [15]. The summary of the applied spectrometric approach for NP surface analysis is presented in Fig. 4.

During exposure to biological and environmental fluids, the NP surface may adsorb many elements and biomolecules, mainly proteins, resulting in protein corona formation onto NP surface. The most frequently used analytical methods to identify and quantify molecules present onto NP surface are based on mass spectrometry coupled with separation techniques. A number of qualitative and quantitative techniques were reported as highly efficient techniques for assessment and evaluation of the dynamics of corona formation over NPs [95]. In turn, molecular mass spectrometric techniques such as electrospray ionization mass spectrometry (ESI-MS) or electrospray ionization tandem mass spectrometry (ESI-MS/MS) and laser desorption/ionization (LDI) and matrix-assisted (MALDI) TOF-MS provided information about the mass of the ligands present onto NP surface [95, 96]. Generally, MS/MS analysis allowed identification of the ligands present on the surface of NPs and an understanding of how these ligands can be linked or interact with particle surface. Although TOF-MS-based approaches were applied for the analysis of the surface chemistry of NPs functionalized with synthetic ligands, the scope of the method was limited to ligands with molecular weight up to 1000 Da [97, 98]. To overcome this drawback and the limitation in terms of mass resolution, ultrahigh-resolution Fourier transform ion cyclotron resonance MS and a combination of LDI and MALDI were proposed [99]. The developed protocol enabled detailed structural characterization of peptide and carbohydrate functionalized AuNPs. On the other hand, in order to determine the presence and composition of protein corona, NP-protein corona fraction was isolated in vitro from a biological fluid and then subjected to the analysis by hyphenated techniques. Recently, the fibrinogen-enriched corona composition onto silica NPs was evaluated by sodium dodecyl sulfate polyacrylamide gel electrophoresis and proteomic/proteoglycomic analysis using liquid chromatography ESI-MS/MS. Based on the proteomic and glycomic fingerprints of plasma fibrinogen, the potential distinction lung cancer patients form controls was found. The proposed method enabledthe evaluation of the changes in fibrinogen that are associated with chronic diseases and provides a new perspective on the application of protein corona in biomarker discovery [100]. Nevertheless, it should be noted that the biological fluids such as blood are very complex due to containing more than 10,000 proteins whose concentrations vary by more than 10 orders of magnitude in protein corona [100, 101]. The typical limit of dynamic range of MS techniques is lower, which leadto protein information loss concerning low-abundance proteins [100]. Thus, the complexicity of real samples is still challenging in protein corona analysis. In the recent study, the investigation of the chemical composition and size distribution of SeNP-HSA conjugates by SP-MIP-OES was reported. The results confirmed corona protein formation on NP surface. However, the proposed technique requires NPs in nanopowder form [75].

For the characterization of NP surface and their interaction with biomolecules, Biological Surface Adsorption Index (BSAI) can be applied [102–107]. The BSAI was developed to identify interactions that occur between NPs and biomolecules which adsorb onto NP surface such as organic compounds, peptides, and proteins. The BSAI-based approach consisted in measuring the forces of surface adsorption in a simulated biological system using a set of compounds with different physicochemical properties, so that it was possible to obtain five nanodescriptors representing the molecular forces responsible for the interaction of a nanomaterial with biomolecules: hydrophobicity, hydrogen bonding, polarity, polarizability, and lone-pair electron. The contribution of each type of molecular interaction in adsorption was determined experimentally on the basis of the degree of adsorption of various organic compounds with diverse structural properties, in the probe sample. The probe sample is the mixture of organic probe compounds characterized by different properties resulting in various interactions forces with NP surface, such as chlorobenzene, phenol, and pyrene [108]. By measuring of the concentration of probe compounds adsorbed onto NP surface and using an appropriate predictive model, the nanomaterial adsorption coefficient was determined. Then, nanodescriptors were created by means of multiple linear regression analysis based on previously calculated adsorption coefficients. By modeling the adsorption behavior of these probes, it was possible to predict the adsorption of small molecules onto different nanomaterials. In mathematical calculations, nanodescriptors were crucial in representing the contributions and relative strengths of each molecular interaction for creating pharmacokinetic and nanomaterial safety assessment model. This predictive model could be applied in biological and environmental systems, for example, nanomedicine, to predict the risk assessment and safety of nanomaterials. To determine the concentration changes of probe components before and after adsorption with nanomaterials, solid-phase microextraction (SPME) coupled to gas chromatography mass spectrometry (GC–MS) allowing a high-throughput analysis was employed [107–109]. This technique enabled simple quantification of organic compounds in different matrices based on their adsorption onto different types of fiber. Due to selective extraction of the target compounds which were present in a free form (not adsorbed into NP surface) in the sample solution, SPME technique did not require the removal of NPs before analysis and enables the analysis directly into reaction vessel, with a reduced effect on the interaction between NPs and probe compounds. The removal of NPs from sample solution before analysis could affect the result of the adsorption due to the release of the probe compounds adsorbed onto NP surface. Based on these results, Xia et al. proposed an index that could be used to characterize NP surfaces and to predict their adsorption properties [103, 109]. On the other hand, Omanović-Mikličanin et al. investigated the various aspects of the SPME-GC-MS technique for characterization of Au and silica NP (SiO_2_ NP) surfaces [108]. They showed differences in behavior between the two type of NPs due to the different surface chemistry. However, the adsorption of NPs on the SPME fiber, kinetic of adsorption of the probe compounds on NPs, and concentration of both NPs and probe compounds were found to be crucial parameters for the SPME analysis of NP-analyte interaction. The presence of both NPs and nanoparticulate-bonded analyte forms on the fiber surface could affect adsorption behavior of the probe compounds. The partition of nanoparticulate-bonded analyte present in the sample solution into the extracting fiber coating could led to overestimation of the concentration of free probe compounds due to entering of its nanoparticulate complex species to the solid phase, which were measured together with the free analyte. On the other hand, the association between various sample components and the probe compounds generally leads to changes in partition coefficients and extraction kinetics; thus, the original partition equilibrium between the bound and the free fractions was changed [110]. Moreover, when the free form is absorbed onto SPME fiber, in the heterogeneous environmental and biological samples containing associated molecules such as proteins, the presence of different sample constituents leads to lowering of the SPME measured concentration [111]. It should be mentioned here that NPs can detoxify many heavy metals, such as cadmium, chromium, and mercury [112]. Thus, NPs can be used for adsorption of toxic metals in aqueous solutions and human body at low cost, easy handling characteristics, and high efficacy. Next to AAS [113], ICP-OES [114], and ICP-MS [115], PCVG coupled with SPME and MIP-OES were applied for monitoring of bioaccessible fraction mercury during their incubation in simulated body fluid in the presence of SeNPs examined as a potential mercury detoxifying agent [116]. Although these techniques enabled determination of both bonded and free form of analytes, they require removal of analyte bonded to NP surface from the analyzed samples, which affects the equilibrium between NP and analyte.

## Conclusion and perspective

Spectrometric techniques are some of the most frequently applied for the identification and characterization of NPs concerning their synthesis yield, particle size and particle number concentration, chemical composition, and surface activity. MS-based techniques are considered as essential tools in NP analysis due to the ability to detect, quantify, and characterize particles in environmentally and biologically relevant conditions. However, the better resolution and higher sensitivities of MS instrumentation as well as more reproducible methodologies to achieve more reliable results are still expected. Although MS-based techniques working in SP mode are considered as the most favorite tools for complete NP characterization, those based on OES detection system show very promising results particularly for NPs’ elemental composition determination and their surface analysis. In terms of methods’ reproducibility and their trueness, the development of validated methods for quantitative NP detection and characterization in biological and environmental samples needs to be investigated. The lack of certified reference materials for NPs with, for example, different NP sizes which influence the calculation of transport efficiency in SP mode of spectrometric methods limits investigation of the reliable analytical protocols for complete NP characterization. In terms of methodology and metrology, NP is usually treated as an analyte or one of its forms of species. The analysis of NP may be performed by different analytical approaches. Literature reports emphasize that depending on the chosen methodology, the physicochemical properties of NPs may vary. Therefore, applying more than one analytical method in one study should be considered to improve the reliability of the results.

However, each analytical techniques offers its strengths and limitations as well; thus, concerning the choice of appropriate analytical approach for the reliable characterization of NPs, there are multiple open questions that need further consideration. The awareness of the fundamental properties of NPs is crucial to investigate NP behavior in the different media and their interaction with environment and organism-environment systems.

## Acronyms

AAS: Atomic absorption spectrometry
AF4: Asymmetrical flow field-flow fractionation
AgNPs: Silver nanoparticles
Ag-Au NPs: Silver-gold bimetallic core-shell nanoparticles
AuNPs: Gold nanoparticles
BSAI: Biological Surface Adsorption Index
CeO_2_ NPs: Cerium nanoparticles
ESI-MS: Electrospray ionization mass spectrometry
ESI-MS/MS: Electrospray ionization tandem mass spectrometry
FFF: Flow field-flow
GC-MS: Gas chromatography mass spectrometry
HSA: Human serum albumin
ICP-MS: Inductively coupled plasma mass spectrometry
ICP-OES: Inductively coupled plasma optical emission spectrometry
ICP-TOF-MS: Inductively coupled plasma time-of-flight mass spectrometry
IEC: Ion exchange column
LDI: Laser desorption/ionization
LOD: Limit of detection
MALDI: Matrix-assisted desorption/ionization
MC-ICP-MS: Multi-collector inductively coupled plasma mass spectrometry
MIP-OES: Microwave plasma optical emission spectrometry
NP: Nanoparticle
NPs: Nanoparticles
PCVG: Photochemical vapor generation
PtNPs: Platinum nanoparticles
RF: Radio frequency
SeNPs: Selenium nanoparticles
Se^0^S^0^ NPs: Sulfur-selenium nanoparticles
SiO_2_ NPs: Silica nanoparticles
SP-ICP-MS: Single-particle inductively coupled plasma mass spectrometry
SP-ICP-QMS: Single-particle inductively coupled plasma quadrupole mass spectrometry
SP-ICP-TOF-MS: Single-particle inductively coupled plasma time-of-flight mass spectrometry
SPIONs: Superparamagnetic iron oxide nanoparticles
SPME: Solid-phase microextraction
SP-MIP-OES: Microwave plasma optical emission spectrometry operating in a single-particle mode
TEM: Transmission electron microscopy
TiO_2_ NPs: Titanium dioxide nanoparticles
TMAH: Tetramethylammonium hydroxide
ZnO NPs: Zinc nanoparticles
